# Application of high‐dose‐rate endorectal brachytherapy in the treatment of locally advanced rectal cancer

**DOI:** 10.1002/pro6.70004

**Published:** 2025-03-30

**Authors:** Tianyao Wang, Yifan Tao, Guanghui Gan, Long Chen, Yuan Xu, Fei Sun, Xiaoting Xu

**Affiliations:** ^1^ First Affiliated Hospital of Soochow University Suzhou China

**Keywords:** High‐dose‐rate Brachytherapy, Rectal cancer, Systematic review

## Abstract

**Purpose:**

This study evaluates the efficacy, toxicity, and survival impact of high‐dose‐rate endorectal brachytherapy (HDR‐EBT) as neoadjuvant therapy for locally advanced rectal cancer.

**Methods:**

A review of 16 studies from PubMed, Embase, and Web of Science (1990–2023) was conducted.

**Results:**

Patients treated with HDR‐EBT alone had a pathological complete response (pCR) rate of 23.7%–35.3% (mean: 24.3%), anal preservation rate of 12.2%–74.9% (mean: 41.8%), and 5‐year progression‐free survival rate of 64.6%–65.4% (mean: 65.3%). When combined with concurrent long‐term radiotherapy and chemotherapy, pCR rates improved from 18.1%–55.0% (mean: 31.0%), with anal preservation rates of 39.6%–51.4% (mean: 45.3%). However, overall survival did not significantly improve.

**Conclusion:**

Integrating advanced techniques such as intensity‐modulated radiation therapy (IMRT) with HDR‐EBT shows promise. This approach particularly benefits patients ineligible for surgery or those adopting a watch‐and‐wait strategy after complete clinical remission, thus highlighting the potential of HDR‐EBT in this treatment landscape.

## Graphical abstract

This image compares the studies included in this article with the meta‐analysis from the Cochrane Library, indicating that the incorporation of brachytherapy in neoadjuvant treatment for rectal cancer can achieve better pathological response rates. Additionally, the use of brachytherapy can reduce the local recurrence rate in patients. However, there are no significant differences in long‐term survival and distant metastasis.

## 1 INTRODUCTION

Colorectal cancer (CRC) is the third most prevalent cancer worldwide and the second leading cause of cancer‐related deaths. In 2020, Global Cancer Observatory (GLOBOCAN) reported 1.9316 million new CRC cases and 935,000 deaths globally, accounting for 10.0% and 9.4% of total cancer incidence and cancer‐related mortality, respectively. Of these, 732,000 new cases were rectal cancer.^1^ Neoadjuvant chemoradiotherapy (NCRT) is currently the standard treatment for locally advanced rectal cancer.

Several challenges exist in treating locally advanced rectal cancer. Liao et al. reported a pathological complete response (pCR) rate of 22.7% with total neoadjuvant therapy (TNT),^2^ indicating that many patients have residual tumors after external beam radiation in neoadjuvant therapy. Brachytherapy is commonly used to enhance the local tumor dose in the curative treatment of cervical cancer. In some parts of Europe, it is used for rectal cancer patients with residual tumors, those who cannot undergo surgery, or those unwilling to do so. Therefore, brachytherapy can be considered as a viable treatment option to improve local tumor control.

Brachytherapy involves placing a source applicator near or within the tumor or postoperative tumor bed. A key advantage of brachytherapy is its ability to deliver high radiation doses to the tumor while minimizing exposure to the surrounding normal tissues.^3^ The physical properties of radioactive isotopes determine their dose rates. These include high dose rate (HDR, >12 Gy/h), medium dose rate (MDR, 2 Gy/h–12 Gy/h), low dose rate (LDR, 0.4–2 Gy/h), or very low dose rate (vLDR, <0.4 Gy/h). Currently, the most commonly used radioactive isotope is high‐dose‐rate iridium‐192.^4^ HDR brachytherapy offers the advantage of shorter treatment duration than LDRs. Common applications of brachytherapy include treating skin, cervical, endometrial, prostate, breast, and rectal cancers. Although brachytherapy for rectal cancer is less common in China and the United States, its use is increasing in Europe.^5^ Brachytherapy can be used as a standalone treatment, typically administered at 26 Gy in four fractions. When used as an adjunct to or following external beam radiation, it is generally administered at 5–10 Gy.

In 2017, Buckley^6^ published a systematic review of HDR‐EBT for operable rectal cancer. This review, published in the *International Journal of Biological Physics*, included clinical studies published between January 1990 and December 2016. It reported the pCR rate, overall survival (OS), progression‐free survival (PFS), surgical complications, and adverse effects of HDR treatment. Its limitations include the lack of detailed toxicity reports, variations in clinical endpoints across trial groups, and the predominance of single‐arm, single‐center, and small‐scale studies. Heterogeneities were observed in patient selection, dose and fractionation of HDR‐EBT, combination with CRT, chemotherapy regimens, and the interval between radiation and surgery. Additionally, the review included studies on HDR‐EBT for operable rectal cancer between January 2017 and December 2023, which offer insights into current treatment approaches.

## 2 METHODS AND MATERIALS

### 2.1 Inclusion and exclusion criteria

1. Study type: Included experimental trials that used HDR‐EBT as neoadjuvant therapy for locally advanced rectal cancer before surgery. Blinding or allocation concealment was not required, and studies had to be published in English.
2. Study participants: Patients >18 years with stage II/III, predominantly T3, operable rectal tumors who received preoperative HDR brachytherapy followed by surgery. Nationality and ethnicity were not restricted.
3. Interventions: The experimental group received either standalone HDR brachytherapy or brachytherapy combined with conventional external beam radiochemotherapy. The control group received conventional NCRT, with no radiation doses and chemotherapy regimens not restricted.
4. Outcome measures included (1) pCR rate, (2) OS, (3) PFS, (4) R0 resection rate, (5) sphincter preservation rate, (6) postoperative complications, (7) Grade 3 adverse reaction rate, and (8) local recurrence and distant metastasis.
5. Exclusion criteria included ①non‐English literature, ②duplicate publications, ③studies that did not report relevant outcome measures, and ④ studies that additionally used targeted therapy.

### 2.2 Search strategy

**FIGURE 1:**
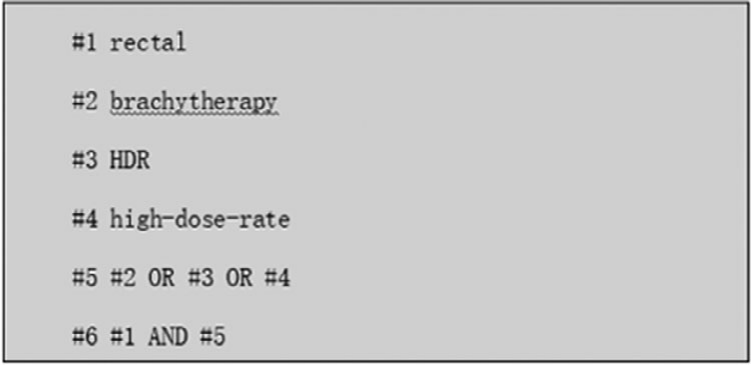
Search Strategy.

Following the 2020 Preferred Reporting Items for Systematic Reviews and Meta‐Analyses (PRISMA) guidelines, computerized searches were conducted in PubMed, Embase, and Web of Science for studies on HDR brachytherapy for rectal cancer.^7^ The search terms included *rectal cancer*, *brachytherapy*, *HDR*, and *high‐dose‐rate*, with studies published between January 2000 and December 2023. Relevant references, systematic reviews, meta‐analyses, and related studies were also examined. The search strategy, with PubMed as an example, is illustrated in Figure 1.

The patient populations included stagesT2–T4, with T3 being the predominant stage, and sample sizes ranged 17–483. Considering that only four randomized controlled trials (RCTs) with inconsistent control methods were available, only studies in which the intervention involved HDR‐EBT were included. In the intervention group, standalone HDREBT was typically administered at 26 Gy/4 fractions, although one study used doses ranging 16–80 Gy/4 fractions.^8^ For HDR‐EBT combined with long‐term chemoradiotherapy, doses of 45–60 Gy were administered in 25–30 fractions, followed by 5–10 Gy in 1–2 fractions. Table 1 presents the characteristics of the included studies, whereas Tables 2 and 3 detail the risk of bias results.

**TABLE 1:** Basic information of studies.

Study ID	Design	No. of patients receiving HDR, n	Treatment details	Outcome indicators
Jakobsen, et al.2006	T3Nx	48	60Gy/30f+5Gy/1f Tigafluorouracil300 mg/m2	① ④⑤⑥⑦
Jakobsen, et al.2007	T3、T4	33	60Gy/30f+5Gy/1f Tigafluorouracil300 mg/m2 +Celecoxib400mg bid	① ④⑤⑥
Jakobsen, et al.2012	T3、T4	105	50.4Gy/28f+10Gy/2f Tigafluorouracil300 mg/m2 +L‐leucovorin22.5 mg/d	① ④⑤⑦
Ane L Appelt, et al. 2014	T3、T4, N0	95	50.4Gy/28f+10Gy/2f Tegafur3×100mg/m2	① ②③④⑤⑧
Tunio, et al. 2010	T3、T4 or N+	17	45Gy/25f+5.5‐7Gy*2f Capecitabine825mg/m2 bid	① ⑥⑦
Sun myint, et al. 2010	‐	29	45Gy/25f+10Gy/2f Capecitabine825mg/m2	①②③④⑤⑦
Arefeh, et al. 2023	‐	22	50.40 Gy/28f Capecitabine825mg/m2	① ②③⑤
R. Engineer, et al.2023	‐	79	50Gy/25f+8‐15Gy/2‐3f Capecitabine825mg/m2	① ②③⑤⑧
Breugom, et al. 2015	T3	141	26Gy/4f	② ⑤⑧
Hesselager, et al. 2013	‐	316	26Gy/4f	① ④⑤⑦
Vuong, et al. 2002	T2–T4	47	26Gy/4f	① ④⑦⑧
Vuong, et al. 2007	T2–T4	96	26Gy/4f	① ②③⑤⑥
Vuong, et al. 2015	T2–T4	483	26Gy/4f	②③
Yanagi, et al. 2000	T2–T4 Nx	115	16‐80Gy/4f	①⑤
Rebecca, et al. 2017	II–III	17	26Gy/4f	①
Garant, et al. 2021	T2、T3 Nx	115/60	26Gy/4f +FOLFOX x6+FOLFOX x6 postoperative 26Gy/4f + FOLFOX x12	① ②③⑥⑧

① pCR rate ②OS ③ DFS/PFS ④ R0 resection rate ⑤ Organ preservation rate ⑥ Adverse reaction rate of Grade 3 or above ⑦ Incidence of surgical complications ⑧Relapse and metastasis

**TABLE 2:** Risk of bias assessment(ROB 2.0).

Study ID	Bias arising from the randomization process	Bias from deviations from intended interventions	Bias in the measurement of the outcome	Bias from missing outcome data	Bias in the selection of the reported result	Overall bias
Jakobsen, et al.2012	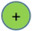	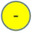	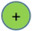	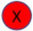	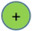	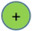
Appelt, et al.2014	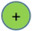	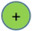	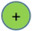	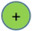	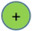	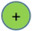
Tunio, et al.2010	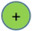	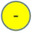	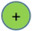	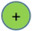	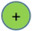	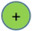
Garant, et al.2021	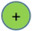	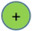	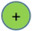	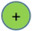	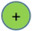	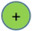

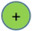
: Low risk. 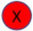: Some concerns. 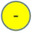: High risk

**TABLE 3:** Risk of bias in non‐randomized studies of interventions(ROBINS‐I).

Study ID	Bias from confounding	Bias in the selection of participants for the study	Bias in the classification of interventions	Bias from deviations from intended interventions	Bias from missing data	Bias in measurement of outcomes	Bias in selection of the reported result	Overall bios
Jakobsen, et al. 2006	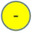	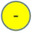	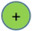	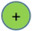	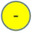	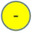	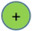	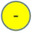
Jakobsen, et al. 2007	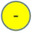	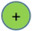	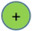	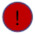	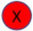	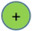	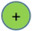	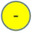
Breugom, et al. 2015	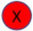	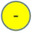	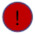	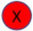	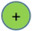	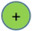	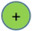	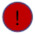
Hesselager, et al. 2013	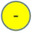	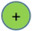	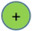	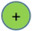	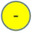	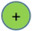	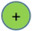	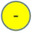
Sunmyint, et al. 2010	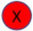	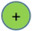	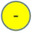	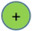	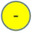	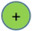	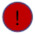	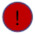
Arefeh, et al. 2023	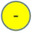	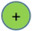	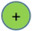	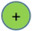	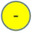	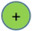	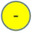	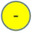
R. Engineer, et al. 2023	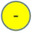	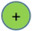	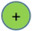	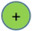	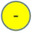	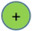	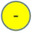	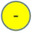
Vuong, et al. 2002	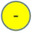	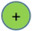	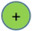	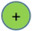	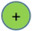	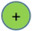	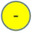	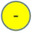
Vuong, et al. 2007	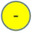	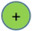	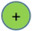	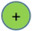	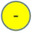	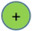	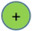	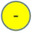
Vuong, et al. 2015	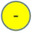	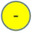	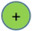	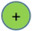	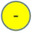	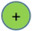	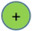	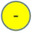
Yanagi, et al. 2000	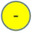	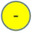	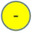	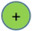	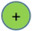	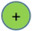	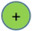	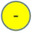
Rebecca, et al. 2017	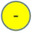	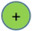	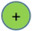	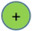	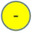	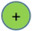	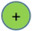	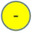

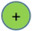
:Low risk 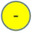:Moderate risk 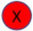:Serious risk 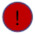:Critical risk

### 2.3 Literature screening and data extraction

Two researchers independently screened the literature and extracted data based on the inclusion and exclusion criteria, and reconciled their findings subsequently. Discrepancies were resolved through discussions with a third party. The extracted information included ①basic details of the included studies, such as title, first author, journal of publication, and publication date; ②basic characteristics of the study participants, including age, sex, and tumor‐node‐metastasis (TNM) tumor staging;③specific intervention details: doses and frequencies of HDR brachytherapy, external beam radiation, chemotherapy, including drug types and dosages; ④outcome measures of interest; and ⑤data relevant to risk of bias assessment.

### 2.4 Bias risk

Two reviewers independently assessed the risk of bias using the Cochrane Handbook guidelines along with the ROB 2.0 and ROBINS‐I scales. The results were compared and discussed, and any disagreements were resolved through consultation with a third researcher. For RCTs, the evaluation items included ①bias from randomization, ②bias from deviations in interventions, ③ measurement bias, ④bias from missing data, ⑤reported bias, and ⑥overall bias. For non‐RCTs, the evaluation items included ①confounding bias,② selection bias, intervention ③classification bias, ④bias from deviations in interventions, bias from missing data, ⑥measurement bias, and ⑦ reporting bias.

## 3 RESULTS

### 3.1 Literature search results

**FIGURE 2:**
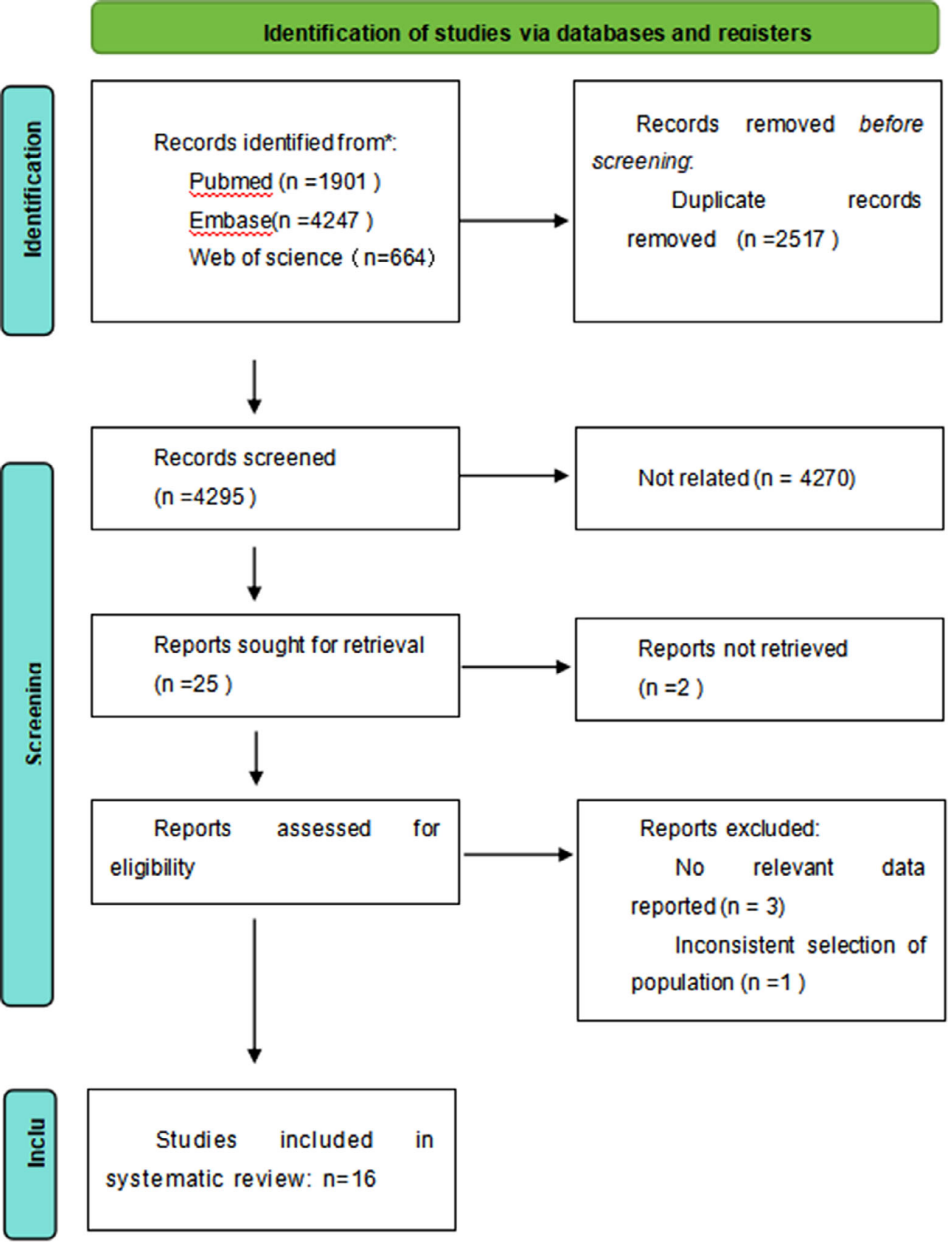
PRISMA flow diagram for study selection.

Overall, 7,457 relevant articles were retrieved. After carefully reviewing titles, abstracts, and full texts, and removing duplicates and irrelevant papers, 24 articles were included. Among these, three articles lacked relevant data, one had population selection issues, three used targeted therapies, and one was a duplicate study. Finally, 16 studies were included in the analysis. The specific process and results of the literature screening are shown in Figure 2.

### 3.2 Basic characteristics and risk of bias assessment of included studies

#### 3.2.1 pCR rate

The pCR rate included in this study was using the Mandard standard (TRG 1), NCCN standard (TRG 0), or pathological results of ypT0N0. Thirteen studies reported pCR rates,^8^, ^9^, ^10^, ^11^, ^12^, ^13^, ^14^, ^15^, ^16^, ^17^, ^18^, ^19^, ^20^, ^21^ with five using the Mandard standard,^10^, ^12^, ^13^, ^15^, ^17^ one using the American Joint Committee on Cancer (AJCC) standard,^21^ one based on the national comprehensive cancer network (NCCN) standard,^18^ two using ypT0N0 as the criterion,^16^, ^19^ three using ypT0 as the criterion,^8^, ^9^, ^14^ and one employing ypT0N0‐2 as the reference.^11^ Among these, eight studies on HDR‐EBT combined with long‐course chemoradiotherapy, with a pCR rates ranging from 18.1%–55.0% (weighted average: 31.0%).^10^, ^12^, ^13^, ^14^, ^15^, ^17^, ^20^, ^21^ Five studies assessed HDR‐EBT alone as neoadjuvant therapy, with pCR rates ranging from 23.7%– 35.3% (weighted average: 24.3%).^8^, ^9^, ^11^, ^16^, ^18^ One study reported HDR‐EBT combined with folinic acid, fluorouracil and oxaliplatin (FOLFOX) chemotherapy, with an overall pCR rate of 30.0%.^19^ Surgery‐related outcomes are detailed in Table 4.

**TABLE 4:** PCR rate R0 resection rate and organ preservation rate.

Study ID	Treatment details	Number of patients receiving surgery, *n*	Number of pCR patients, *n* (pCR rate)	No. of patients undergoing R0 resection, *n* (R0 rate)	No. of organ reservation, *n* (Organ preservation rate)
Jakobsen, et al. 2006	60Gy/30f+5Gy/1f Uracil‐tegafur300 mg/m2	48	13 (27.1%)	47 (97.9%)	19 (39.6%)
Jakobsen, et al. 2007	60Gy/30f+5Gy/1f Uracil‐tegafur300 mg/m2 + Celexocib400mg bid	33	7 (21.2%)	32 (97.0%)	16 (48.5%)
Jakobsen, et al. 2012	50.4Gy/28f+10Gy/2f Uuracil‐tegafur300 mg/m2 and L‐leucovorin22.5 mg/d	105	19 (18.1%)	87 (87.9%)	54 (51.4%)
Tunio, et al. 2010	45Gy/25f+5.5‐7Gy*2f Capecitabine825mg/m2 bid	17	5 (29.4%)(ypT0)	‐	5 (50.0%)
Sun myint, et al. 2010	45Gy/25f+10Gy/2f Capecitabine825mg/m2 bid	29	9 (31.3%)	24 (82.8%)	18 (62.1%)
Ane L Appelt, et al. 2014	50.4Gy/28f+10Gy/2f Tegafur3×100mg/m2	95	45 (47.4%)	90 (94.7%)	38 (40.0%)
Garant, et al. 2020	26Gy/4f +FOLFOX x6 + FOLFOX x6 postoperative 26Gy/4f + FOLFOX x12	115/60	35 (30.4%)	‐	‐
Arefeh, et al. 2023	50.4Gy/28f+8Gy/2f Capecitabine825mg/m2 bid	20	11 (55.0%)	‐	11 (55.0%)
R. Engineer, et al. 2023	50Gy/25f+8‐15Gy/2‐3f Capecitabine825mg/m2 bid	79	21 (28.8%)	‐	36 (45.6%)
Yanagi, et al. 2000	16‐80Gy/4f	115	12 (10.4%)(ypT0)	‐	14 (12.2%)
Vuong, et al. 2002	26Gy/4f	47	15 (31.9%)(ypT0)	46 (97.9%)	‐
Vuong, et al. 2007	26Gy/4f	96	28 (29.2%)(ypT0N0‐2)	‐	45 (46.9%)
Hesselager, et al. 2013	26Gy/4f	316	75 (23.7%)	265 (83.9%)	‐
Breugom, et al. 2015	26Gy/4f	141	‐	‐	49 (34.8%)
Rebecca, et al. 2017	26Gy/4f	17	6 (35.3%)	‐	‐

#### 3.2.2 R0 resection rate

A total of 7 studies reported R0 resection rates.^9^, ^10^, ^12^, ^13^, ^15^, ^16^, ^17^ Among these, five studies involved HDR‐EBT combined with long‐course chemoradiotherapy, with R0 resection rates ranging from 82.8% to 97.9% (weighted average rate of 92.1%).^10^, ^12^, ^13^, ^15^, ^17^ Two studies assessed HDR‐EBT alone as neoadjuvant therapy, with an overall R0 resection rate ranging from 83.9% to 97.9% (weighted average rate of 85.7%).^9^, ^16^

#### 3.2.3 Anal preservation rate

A total of 11 studies reported anal preservation rates.^8^, ^10^, ^11^, ^12^, ^13^, ^14^, ^15^, ^17^, ^20^, ^21^, ^22^ Among these, eight studies involved HDR‐EBT combined with long‐course chemoradiotherapy, with anal preservation rates ranging from 39.6%–51.4% (weighted average rate of 45.3%).^10^, ^12^, ^13^, ^14^, ^15^, ^17^, ^20^, ^21^ Three studies assessed HDR‐EBT alone as neoadjuvant therapy, with anal preservation rates ranging from 12.2% to 74.9% (weighted average rate of 41.8%).^8^, ^11^, ^22^

#### 3.2.4 PFS and OS

Overall eight studies were reported on PFS and OS.^11^, ^13^, ^17^, ^19^, ^20^, ^21^, ^22^, ^23^ Four studies reported on the prognosis of HDR‐EBT combined with long‐course chemoradiotherapy.^13^, ^17^, ^20^, ^21^ In two studies published before 2023, the 2‐year PFS and OS rates were 65.5%, 68.4%, 72.4%, and 84.2%, respectively.^13^, ^17^ One study reported the 5‐year outcomes with a PFS of 51.6% and OS of 64.2%.^17^ Three studies reported results for HDR‐EBT alone as neoadjuvant therapy, with a 5‐year PFS ranging from 64.6% to 65.4% (weighted average rate of 65.3%) and a 5‐year OS ranging from 70.0% to 88.7% (weighted average rate of 75.6%).^11^, ^22^, ^23^ Garant et al.^19^ reported a 5‐year PFS of 69.7% and OS of 82.9% for HDR‐EBT combined with FOLFOX. The results of these studies are presented in Table 5.

**TABLE 5:** Recurrence and survival after HDR‐EBT for operable rectal cancer.

Study	Treatment details	No. of patients, *n*	Median follow‐up time, *m*	DFS/PFS, *n*	OS, *n*
Sun myint, et al. 2010	45Gy/25f+10Gy/2f Capecitabine825mg/m2	29	17	2‐y PFS 19 (65.5%)	2‐y OS 21 (72.4%)
Ane L Appelt, et al. 2014	50.4Gy/28f+10Gy/2f Tegafur3×100mg/m2	95	65	2‐ PFS 65 (68.4%) 5年PFS 49 (51.6%)	2‐y OS 80 (84.2%) 5‐y OS 61 (64.2%)
Arefeh, et al. 2023	50.4Gy/28f+8Gy/2f Capecitabine825mg/m2 bid	22	99	8‐y PFS 17 (77.2%)	8‐y OS 19 (86.4%)
R. Engineer, et al. 2023	50Gy/25f+8‐15Gy/2‐3f Capecitabine825mg/m2 bid	79	30	3‐y PFS 70 (88.6%)	3‐y OS 77 (97.5%)
Breugom, et al. 2015	26Gy/4f	141	79	‐	5‐y OS 125 (88.7%)
Vuong, et al. 2007	26Gy/4f	96	60	5‐y DFS 62 (64.6%)	5‐y OS 67 (70.0%)
Vuong, et al. 2015	26Gy/4f	483	63	5‐y DFS 316 (65.4%)	5‐y OS 352 (72.9%)
Garant, et al. 2020	26Gy/4f+FOLFOX x6+FOLFOX x6 postoperative 26Gy/4f +FOLFOX x12	115/60	48	5‐y DFS 79 (68.7%)/43(71.7%)	5‐y OS 96 (83.5%)/49(81.7%)

#### 3.2.5 Adverse reaction events of grade 3 or above

Five studies reported the incidence of grade 3 adverse events.^10^, ^11^, ^12^, ^14^, ^19^ Only one study reported adverse events for HDR‐EBT treatment alone, of 100 patients, 99 had grade 2 proctitis, 1 had grade 3 proctitis, and 2 developed grade 3 dermatitis. No other adverse events were reported.^11^ Three studies reported the results of long‐course radiotherapy combined with HDR‐EBT.^10^, ^12^, ^14^ In these studies, 16 patients (n = 98) experienced grade 3 adverse events (16.3%), with diarrhea (n = 11) and pain (n = 12) being the most common. Another study reported that HDR‐EBT combined with FOLFOX chemotherapy documented a total of 43 grade 3 adverse events (24.6%), including gastrointestinal reactions in six patients, hematological toxicity in 13 patients, vascular disorders in eight patients, and cardiac disorders in five patients.^19^

#### 3.2.6 Surgical‐related complications

A total of 5 studies reported on surgical‐related complications.^9^, ^10^, ^13^, ^15^, ^16^ Among these, 3 studies reported results for long‐course radiotherapy combined with HDR‐EBT.^10^, ^13^, ^15^ In these studies, 48 patients (n = 182) experienced surgery‐related complications (26.4%), including wound infections in 21 patients (11.53%), reoperation in nine patients (5.95%), death in one patient (0.55%), bowel obstruction in four patients (2.20%), fistula formation in one patient (0.55%), urinary system dysfunction in four patients (2.20%), and other possible complications in eight patients (4.40%). Only 2 studies have reported surgery‐related complications with HDR brachytherapy alone.^9^, ^16^ A total of 58 patients (n = 363) experienced complications (16.0%), including wound infections in three patients (0.83%), reoperation in 13 patients (3.58%), death in four patients (1.10%), fistula formation in two patients (0.55%), urinary system dysfunction in four patients (1.10%), cardiovascular complications in 30 patients (8.26%), and other possibly related complications in two patients (0.55%).

#### 3.2.7 Local recurrence and distant metastasis

Five studies reported local recurrence and distant metastases.^9^, ^17^, ^19^, ^20^, ^22^ In HDR‐related studies, of 188 patients, seven experienced local recurrence, and five experienced distant metastasis. In the long‐course radiotherapy combined with the HDR‐EBT treatment group, 12 of 174 patients had local recurrence and 35 had distant metastasis. In the HDR‐EBT combined with chemotherapy group, 10 patients experienced local recurrence and 38 patients experienced distant metastasis.

## 4 DISCUSSION

According to the 2023 Chinese Guidelines for the Diagnosis and Treatment of CRC, neoadjuvant therapy for rectal cancer is primarily suitable for patients with resectable T3, N+, or T4 rectal cancer with the main methods being chemoradiotherapy (CRT), short‐course radiotherapy combined with consolidation chemotherapy (SCRT+CCT), and TNT. Brachytherapy has not been previously described.^24^ This systematic review mainly investigated the application of HDR‐EBT in the neoadjuvant treatment of rectal cancer.

This systematic review included studies that used HDR‐EBT as neoadjuvant therapy for rectal cancer. The results of two meta‐analyses from the Cochrane Library were compared to determine the direct differences between HDR‐EBT and conventional CRT.^2^, ^25^ Table 6 presents the results.

**TABLE 6:** Comparison of HDR‐EBT and Cochrane Meta‐analysis Results.

Treatment measures	pCR rate	5‐y DFS/PFS	5‐y OS	R0 resection rate	Organ preservation rate	Adverse reaction rate of grade 3 or above		Postoperative complication rate	Rate of local recurrence	Rate of distant metastasis
HDR‐EBT	23.7%	65.3%	75.6%	85.7%	41.8%	‐		16.0%	3.7%	2.7%
HDR‐EBT and CRT	31.0%	63.3%	76.3%	92.1%	45.3%	16.3%		27.5%	6.9%	20.1%
HDR‐EBT and chemotherapy	29.7%	69.7%	82.9%	‐	‐	33.7%		‐	5.7%	21.7%
Zhang, et al. 2022										
CRT	15.2%	61.2%	74.5%	86.7%	54.2%	22.6%		30.1%	9.5%	24.3%
SCRT	15.6%	65.0%	78.1%	89.4%	59.6%	28.3%		32.9%	11.1%	21.7%
Liao, et al. 2022										
CRT	13.6%	‐	‐	84.6%	‐		27.6%	21.2%	9.6%	24.2%
TNT	22.7%	‐	‐	86.5%	‐		28.9%	21.0%	12.2%	20.0%

The results indicate that HDR‐EBT provides better surgical outcomes than traditional NCRT. Specifically, for the pCR rate, the odds ratios (OR) for radiotherapy combined with HDR‐EBT versus traditional CRT and TNT were 2.28 and 1.37, respectively. Regarding the anal preservation rate, the OR for radiotherapy combined with HDR‐EBT versus traditional CRT was 1.85. Relevant RCTs from studies by Jakobsen^15^ and Appelt^17^ have confirmed these findings. However, owing to limitations in sample sizes and variations in chemotherapy regimens, meta‐analyses have not yielded consistent results. In studies using different dosing separation schemes, we conducted further research on CRT combined with HDR‐EBT based on the equivalent biological dose of different radiotherapy schemes. This study showed that an increase in the equivalent biological dose did not lead to an improvement in the pCR rate (p = 0.685). This may have been due to the small sample size. Therefore, we look forward to further studies that provide suitable brachytherapy schemes. Currently, observation and watchful waiting are options for some patients who meet the clinical complete response (cCR) criteria after NCRT, thus avoiding surgery. We hope to achieve cCR with HDR‐EBT in patients who do not meet the CR criteria, thereby avoiding surgery and reducing postoperative complications, such as wound infections, anastomotic leaks, and bladder dysfunction.

In the present study, patients treated with HDR‐EBT had a higher rate of anal preservation than those treated with CRT. Anal preservation helps improve the quality of life of patients with rectal cancer. Given that the included studies ranged from 2000 to 2023, some did not describe the distance of the tumor from the anal margin, and considering the increasing emphasis on anal preservation by surgeons and patients, these benefits should be considered as references. Additionally, the ongoing optimization of chemotherapy drugs has improved tumor regression and increased anal preservation rates.

In the OPERA trial conducted by Gerard et al.,^26^ the addition of contact X‐ray brachytherapy significantly improved cCR and anal preservation rates for early cT2‐cT3 patients, with anal preservation rates exceeding 90% for tumors less than 3 cm in diameter. Contact X‐ray brachytherapy (Papillon technique) delivers surface 50 kV photons directly to the tumor to increase the dose around the tumor. However, this treatment mode has a rapid dose fall‐off; while the dose at the tumor surface is 100%, it drops to 45% at 5 mm and 20% at 10 mm. Therefore, the Papillon technique is not suitable for deeply infiltrated bulky residual tumors but is better suited for small exophytic residual tumors. HDR‐EBT, particularly three‐dimensional image‐guided brachytherapy, can increase the radiation dose to the residual primary tumors, covering more than 80% of the tumor after external beam radiotherapy. If residual tumors deeper than 10 mm are detected on rectal ultrasonography or posttreatment MRI, CT within the planned treatment area is recommended for adequate tumor coverage.

These results confirm that HDR‐EBT improves local control, but has no significant impact on distant metastasis rates. We believe that TNT can be widely used for the treatment of locally advanced rectal cancer. We recommend performing close‐range radiotherapy during consolidation chemotherapy, although this depends on the patient's tumor regression and tolerance.

Research has indicated that pCR after surgery predicts a better prognosis.^27^ Several studies have shown no significant differences in the PFS or OS between patients receiving HDR‐EBT and those receiving traditional long‐course chemoradiotherapy or 5 × 5 Gy short‐course radiotherapy. However, two studies published in 2023 found that using IMRT combined with HDR as a neoadjuvant regimen resulted in better survival rates than traditional CRT or TNT.^20^, ^21^ Owing to the limited number of studies and sample sizes, this regimen requires further exploration.

The incidence of grade 3 or higher adverse events with HDR‐EBT combined with chemoradiotherapy was notably lower than that with traditional chemoradiotherapy or TNT. This may be due to the smaller irradiation field of HDR‐EBT and differences in chemotherapy regimens, with traditional regimens like FOLFOX, FOLFIRI, and XELOX causing more hematological toxicity and diarrhea, while HDR‐EBT primarily causes abdominal pain and diarrhea. In the MORPHEUS Phase II–III Study, it was shown that patients receiving brachytherapy had an improved PFS compared to those receiving EBRT.^28^ There were no significant differences between the two groups in terms of acute toxicities, postoperative surgical complications, or medical complications within one month after surgery. Therefore, we concluded brachytherapy toxicity was within an acceptable range. Earlier studies did not report adverse events. Therefore, we hope that future studies will provide more detailed reports.

Patients receiving HDR‐EBT had significantly lower local recurrence rates than those treated with traditional preoperative chemoradiotherapy. These findings suggest that HDR‐EBT may enhance local disease control and potentially lead to improved patient outcomes. Despite this advantage in local recurrence, the probability of distant metastasis between the two groups showed no significant differences. This indicates that although HDR‐EBT may effectively target local diseases, it does not necessarily affect systemic disease progression in the same way. Further research is needed to understand the mechanisms underlying these findings and to explore how combining HDR‐EBT with other treatments could optimize overall patient outcomes. By focusing on both local control and distant metastasis, we can better tailor the treatment strategies for patients with rectal cancer.

In future research, we suggest incorporating HDR‐EBT into the TNT process and conducting a randomized controlled trial (RCT) to compare this combined approach with the use of TNT alone for neoadjuvant therapy in rectal cancer. This would allow for a direct comparison of the benefits to patients with the addition of HDR‐EBT. Additionally, MRI, digital rectal examination, and colonoscopy can be used to assess cCR and compare treatment efficacy.

Currently, HDR‐EBT is not extensively used. It is mainly suitable for patients who are generally inoperable or unsuitable for surgery, or for those with residual tumors after external beam radiotherapy (EBRT) and chemotherapy. These patients often require additional doses to achieve tumor control, and HDR‐EBT can minimize the impact on surrounding healthy tissues. Therefore, we recommend HDR‐EBT for supplemental dosing.

Current research on HDR‐EBT combined with long‐course chemoradiotherapy lacks a standardized dosing regime, with studies ranging from 5–15 Gy. This variability highlights the urgent need to establish uniform brachytherapy dosing protocols to improve the quality of future studies and reach a consensus on minimum requirements for study endpoints. Most included studies were nonRCTs; therefore, more RCTs are required to validate these findings. Additionally, the limited sample sizes and absence of long‐term follow‐up data, such as five‐year or ten‐year prognoses, in studies combining HDR‐EBT with long‐course chemoradiotherapy hinder comprehensive assessment of the treatment's long‐term benefits. Future research should aim to provide robust data on long‐term outcomes to determine the efficacy and safety of this therapeutic approach.

Integrating HDR‐EBT with traditional NCRT can improve post‐surgical pCR rates. However, studies indicate this does not significantly improve OS or PFS than CRT alone. Recent studies have suggested that IMRT with HDR‐EBT as a neoadjuvant therapy regimen may improve PFS and OS rates. Specifically, HDR‐EBT‐related adverse events, such as proctitis and grade 1 or 2 acute toxicity, remain within acceptable limits. Further clinical research is warranted to optimize the application, dosing strategies, adverse effect management, and survival outcomes associated with HDR‐EBT.

## CONFLICT OF INTERESTS STATEMENT

The authors declare that there is no conflict of interests.

## ETHICAL STATEMENT

Not applicable.

## Data Availability

Data sharing is not applicable to this article as no new data were created or analyzed in this study.
